# Selective-Area Growth Mechanism of GaN Microrods on a Plateau Patterned Substrate

**DOI:** 10.3390/ma16062462

**Published:** 2023-03-20

**Authors:** Min-joo Ahn, Woo-seop Jeong, Kyu-yeon Shim, Seongho Kang, Hwayoung Kim, Dae-sik Kim, Junggeun Jhin, Jaekyun Kim, Dongjin Byun

**Affiliations:** 1 Department of Materials Science and Engineering, Korea University, 145 Anam-ro, Seongbuk-gu, Seoul 02841, Republic of Korea; 2 Natural Science Research, Korea Advanced Institute of Science and Technology (KAIST), 291 Daehak-ro, Yuseong-gu, Daejeon 34141, Republic of Korea; 3 Advanced View Technology Inc., Ansan 15588, Republic of Korea; 4 Department of Photonics and Nanoelectronics, Hanyang University, Ansan 15588, Republic of Korea

**Keywords:** gallium nitride, GaN, aluminum nitride, AlN, pulsed MOCVD, selective-area growth, SAG, epitaxial growth

## Abstract

This study provides experimental evidence regarding the mechanism of gallium nitride (GaN) selective-area growth (SAG) on a polished plateau-patterned sapphire substrate (PP-PSS), on which aluminum nitride (AlN) buffer layers are deposited under the same deposition conditions. The SAG of GaN was only observed on the plateau region of the PP-PSS, irrespective of the number of growth cycles. Indirect samples deposited on the bare c-plane substrate were prepared to determine the difference between the AlN buffer layers in the plateau region and silicon oxide (${\mathrm{SiO}}_{2}$). The AlN buffer layer in the plateau region exhibited a higher surface energy, and its crystal orientation is indicated by AlN [001]. In contrast, regions other than the plateau region did not exhibit crystallinity and presented lower surface energies. The direct analysis results of PP-PSS using transmission electron microscopy (TEM) and electron backscattered diffraction (EBSD) are similar to the results of the indirect samples. Therefore, under the same conditions, the GaN SAG of the deposited layer is related to crystallinity, crystal orientation, and surface energy.

## 1. Introduction

The microstructure of III-nitride compound semiconductors has attracted a considerable amount of research interest for new applications in photonics, thermocouples, and magnetic fields [1,2]. The reasons for this large amount of attention on the microstructure of III-nitrides include the elimination of the quantum confined stark effect (QCSE) [3,4], the reduction of threading dislocation density [5,6,7], and the reduction of the internal stress in the epi-structure [8,9]. Due to these advantages, the microstructures of III-nitride materials have been widely used in optoelectronics and high-speed electronics, including light-emitting diodes (LEDs) [10,11], energy harvesting applications, and high-electron mobility transistors (HEMT). Particularly, gallium nitride (GaN) has attracted attention as a next-generation semiconductor material owing to its wide band gap (3.41 eV), high electron mobility in the two-dimensional electron gas in the HEMT, and high thermal stability.

Several methods have been developed over the last few decades to grow GaN microstructures epitaxially, such as rods and wires, using metal-organic chemical vapor deposition (MOCVD) [12,13], molecular beam epitaxy (MBE) [14,15,16,17], and hydride vapor phase epitaxy (HVPE) methods. In the case of MOCVD, the development of pulse-mode MOCVD, reported by Hersee et al. in 2006 [18], has led to a breakthrough that enables the growth of GaN microrods. Several groups have studied the mechanism of GaN microrods growth using pulsed MOCVD.

The selective-area growth of GaN is an important technique used for GaN microrod growth [19,20]. A common approach to selectively obtain well-aligned GaN microstructures is to fabricate a hole-patterned template using lithography with a mask. Selective-area growth refers to growth only within the holes, while avoiding growth on the mask, such as titanium (Ti) [21,22], silicon nitride (${\mathrm{SiN}}_{x})$ [23,24], and silicon dioxide (${\mathrm{SiO}}_{2}$) [25,26]. Many patterning methods have been developed to form microscale patterns, such as silica nanosphere patterning [27], porous anodic alumina (PAA) patterning [28], electron beam lithography (EBL) [29,30], nanoimprint [29,31], and photolithography [32,33].

For the selective-area growth of GaN using conventional hole patterning methods [5,25], the layer underneath the mask pattern should include a buffer layer, such as a GaN layer for homogeneous epitaxial growth and aluminum nitride (AlN) layer for heterogeneous epitaxial growth. This is because the opening of the mask serves as a preferred nucleation site for atomic absorption when compared with the surface of the amorphous mask. Thus, the selective-area growth mechanism causes the adatoms of the precursor to escape from the surface of the mask, which is a non-preferential nucleation site, and become trapped at the opening of the mask, which is the preferential nucleation site [34].

Unlike the conventional hole-patterning method, we introduced a polished plateau-patterned sapphire substrate (PP-PSS) as a novel patterning method used to selectively grow GaN microrods in our previous study [35]. PP-PSS is a substrate in which ${\mathrm{SiO}}_{2}$ is deposited on a patterned sapphire substrate (PSS) over the entire surface, and a AlN buffer layer is deposited on the entire surface after exposing a c-plane sapphire without ${\mathrm{SiO}}_{2}$ on the upper part of the PSS lens through polishing. PP-PSS, a patterning method capable of compensating for the limitations of the existing hole patterning method, has the advantages of a short tact time, low-cost process, large-area patterning, and controllable pattern size based on the polishing time [35]. For the process of GaN selective-area growth on the PP-PSS, in which the entire surface of the substrate is deposited with an AlN buffer layer, it has a different structure to the existing hole patterning method, in which the mask site and material site to be deposited are different. Nevertheless, a previous study showed that the GaN microrods can selectively grow, even though the AlN buffer layer was deposited under the same deposition conditions on the PP-PSS. Unlike the GaN selective area growth mechanism on the conventional hole-patterned substrate [34], studies on GaN selective area growth on the AlN layer deposited under the same deposition conditions are required for a more advanced applications.

In this study, we have investigated the selective-area growth of GaN microrods on an AlN buffer layer deposited using PP-PSS using several experiments. Since the system size of the GaN selective-area growth on the PP-PSS is ≤5 μm, the substrate used in the experiment was unpatterned and of two types (AlN/sapphire and AlN/${\mathrm{SiO}}_{2}$/sapphire) to indirectly compare with the PP-PSS. The relationship between the mechanism of GaN selective-area growth and substrate properties such as crystallinity, crystal orientation, and surface energy has been discussed by referring to various measurements obtained using PP-PSS and the indirect substrate samples.

## 2. Materials and Methods

To fabricate the PP-PSS structure, we prepared a two-inch PSS with lenses of 2.45 μm in diameter and 1.55 μm in height. A ${\mathrm{SiO}}_{2}$ layer with a thickness of 100 nm was deposited on the PSS substrate in the chamber atmosphere at 250 °C, using plasma-enhanced chemical vapor deposition (PECVD) equipment. Then, the ${\mathrm{SiO}}_{2}$ deposited PSS substrate was polished for 30 s with a slurry using a chemical-mechanical polishing equipment. The slurry was prepared by mixing 0.02 μm colloidal silica suspention and deionized water in a 1:1 ratio. An AlN buffer layer with a thickness of 20 nm was deposited on PP-PSS via radio frequency sputtering at low pressure at 200 ℃ to alleviate the lattice mismatch between GaN and substrate. The undoped GaN microrods was grown on the PP-PSS at 1020 ℃ with the optimized pulsed-mode growth cycle conditions. The atmosphere inside the MOCVD chamber was injected by a continuous flow of pure hydrogen ($H_{2},\;99.999\;\%$) as the carrier gas, a pulse flow of trimethylgallium (TMGa) and ammonia (${\mathrm{NH}}_{3}$) as Ga and N precursors. The optimized pulse-mode growth cycle consisted of TMGa injection and interruption steps, followed by the injection and interruption steps of ${\mathrm{NH}}_{3},$ for 10, 6, 2, and 2 s, respectively. GaN microrods were grown over 2, 4, 6, 10, and 100 pulsed-mode cycles. All of the above processes are listed in progressive order, and all template sizes after ${\mathrm{SiO}}_{2}$ deposition were cut into squares with dimensions of 1 × 1 cm.

To indirectly confirm the selective-area growth, we used a c-plane sapphire substrate rather than PSS. An AlN layer was deposited on the substrate on which a ${\mathrm{SiO}}_{2}$ layer was deposited (sample I) and on a substrate on which a ${\mathrm{SiO}}_{2}$ layer was not deposited (sample II). The deposition conditions for the ${\mathrm{SiO}}_{2}$ layer were the same as those used for the PP-PSS substrate, and the AlN deposition conditions were identical. In addition, the two samples were heated in an MOCVD chamber under constant pressure to reach the GaN growth temperature, and then immediately cooled. The selective growth of GaN on the PP-PSS s was observed using field-emission scanning electron microscopy (FE-SEM; Hitachi S-4300 (Hitachi, Tokyo, Japan), COXEM CX-200). Contact angle measurements were used to analyze the surface energy and surface roughness, and confirmed using atomic force microscopy (AFM; Park systems NX10, Park Systems, Suwon, Republic of Korea). Advancing contact angles of water and diiodomethane were measured using a goniometer system from KRÜSS GmbH (Hamburg, Germany). The crystalline and crystal orientation properties of the c-plane samples were confirmed using pole figure measurements on a PANalytical X-ray diffractometer (XRD, Malvern PANalytical Empyrean, Malvern Panalytical, Almelo, The Netherlands) operated at 3 kW. The crystalline characteristics of PP-PSS at each position were observed using high-resolution transmission electron microscopy (TEM; FEI Technai F20 G2, FEI, Valley City, ND, USA) operated at 200 kV and electron backscatter diffraction (EBSD; model EDAX Velocity Super, EDAX, Mahwah, NJ, USA) at 15 kV after vibratory polishing during sample preparation.

## 3. Results and Discussion

**Figure 1:**
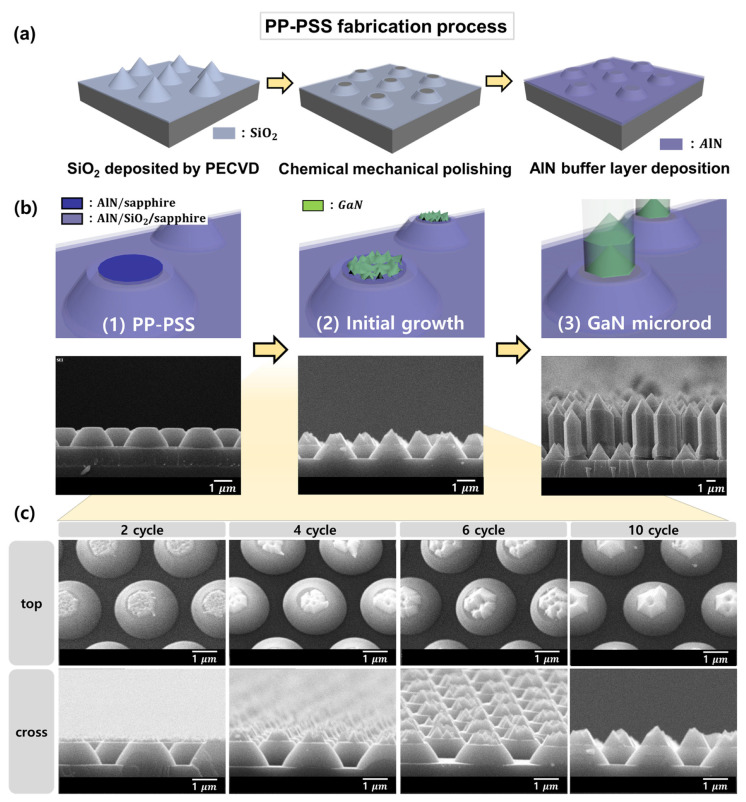
(**a**) A schematic representation of the PP-PSS fabrication process. (**b**) A schematic illustration and cross-sectional SEM image of GaN growth on the PP-PSS. (**c**) The top and cross-sectional SEM image of GaN growth on the PP-PSS depending on the number of growth cycles.

Figure 1 shows the GaN microrods grown on the PP-PSS, and the GaN growth behavior for each growth stage is shown using a schematic illustration and SEM images. Figure 1a shows the fabrication process used for the PP-PSS. In the schematic diagram of the PP-PSS substrate in Figure 1(b1), it can be seen that the flat part of the top of the lens of the PP-PSS is marked in different colors. The AlN buffer layer at the top of the lens was deposited on the sapphire layer and not on the ${\mathrm{SiO}}_{2}$ layer, which is known as the plateau region. Figure 1(b2) shows GaN growth under pulsed-mode growth conditions for 10 cycles. GaN appears to only grow in the plateau region, and was not observed at any other positions. Figure 1(b3) shows the GaN microrods grown under pulsed mode growth conditions over 100 cycles, and most of the microrods were observed to have lengths of ~6 μm. Figure 1c shows the morphology of GaN grown on the PP-PSS using pulsed-mode MOCVD according to the number of cycles observed using SEM. The growth conditions were the same as those used in Figure 1b, with the exception of the cycle count. It was growing for 2, 4, 6, and 10 cycles, and when observing the cross-sectional SEM image it can be clearly seen that the amount of GaN growth increases as the number of cycles increases. Additionally, when confirming the 20° tilt SEM image, even if the amount of GaN growth was small, it grew while maintaining a hexagonal pyramid shape. Referring to Figure 1c, it can be observed that GaN only grew in the plateau region, despite the AlN buffer layer being deposited on the entire surface prior to GaN being grown under the same conditions, and that GaN did not grow in the other regions. This indicates that it is a selective-area growth method that reliably grows in the desired area only.

**Figure 2:**
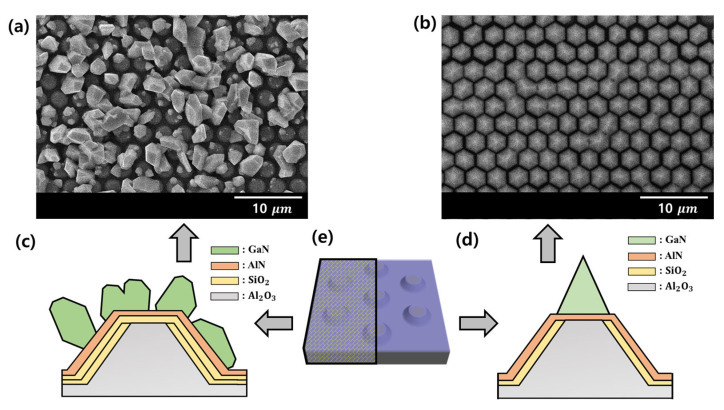
Top-view of the SEM image of GaN growth on the PP-PSS in which AlN was deposited according to the material used as the substrate. As the reference on the plateau region, half of the substrate is (**a**) AlN/${\mathrm{SiO}}_{2}$/sapphire and the other half is (**b**) AlN/ sapphire. (**c**,**d**) A schematic representation of half of the (**e**) PP-PSS and structure of (**a**) AlN/${\mathrm{SiO}}_{2}$/sapphire and (**b**) AlN/sapphire with respect to the plateau region.

Further experiments were conducted to observe the GaN selective-area growth mechanism on the AlN layer of the mask or patterned AlN layer. Figure 2a indicates that ${\mathrm{SiO}}_{2}$ was deposited once more on half of the template area prior to depositing the AlN buffer layer, as shown in Figure 1a, after which the AlN buffer layer was deposited under the same conditions. Therefore, ${\mathrm{SiO}}_{2}$ was deposited under the AlN buffer layer located in the plateau region in Figure 2a, whereas ${\mathrm{SiO}}_{2}$ was not present under the AlN buffer layer of the plateau region in Figure 2b. As a result of growing GaN on the substrate in continuous mode for 10 min, it can be seen from the top-view SEM image in Figure 2a that the orientation of GaN was grown in all regions with a non-tendency or disordered form. Contrastingly, Figure 2b shows that GaN with a hexagonal horn morphology was only formed in the plateau region. It was determined that GaN with a hexagonal pyramid shape was grown in a form that maintained the order. As illustrated in Figure 2a, it can be confirmed that the nucleation site was also observed in AlN grown on ${\mathrm{SiO}}_{2}$. Additionally, the overall AlN is a nucleation site, but it can be confirmed that GaN was grown in the only plateau region target for growth (Figure 2b).

**Figure 3:**
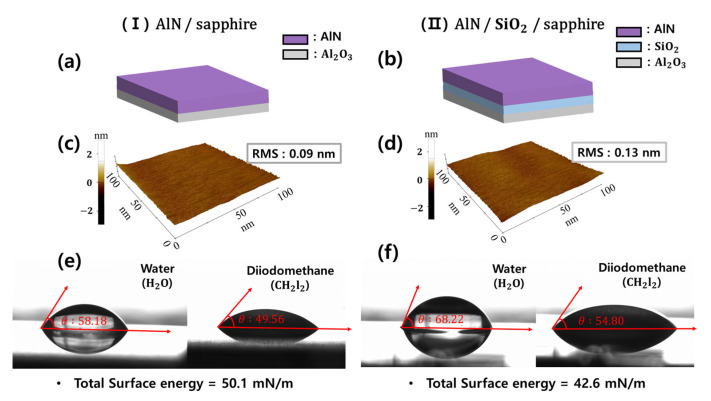
(**a**,**b**) A schematic representation of the structure of the indirect samples: (I) AlN/sapphire, (II) AlN/${\mathrm{SiO}}_{2}$/sapphire. (**c**,**d**) Surface roughness measurements with two indirect samples using AFM. (**e**,**f**) Contact angles of water and diiodomethane on the two indirect samples: (I) and (II).

Figure 1c shows that the size of the plateau region was observed to be ~1 μm. As this measurement was small and the patterns were located separately, it was difficult to selectively analyze the surface characteristics of the plateau and other regions. Figure 3a,b show the two templates with different materials deposited on the planar c-plane sapphire substrate prepared to analyze the selective-area growth phenomenon using the same deposition conditions used for the 20 nm AlN buffer layer: (I) AlN/sapphire and (II) AlN/${\mathrm{SiO}}_{2}$/sapphire. Additionally, the samples were prepared to observe the surface characteristics of the AlN buffer layer prior to the epitaxial growth of GaN. They were heated to the GaN growth temperature under a constant pressure atmosphere, and then immediately cooled. The AlN-deposited sample without the ${\mathrm{SiO}}_{2}$ layer, sample (I), was indirectly similar to the plateau region in the PP-PSS. Additionally, the sample with AlN deposited after ${\mathrm{SiO}}_{2}$ layer deposition, sample (II), is indirectly similar to the region of the c-plane with the exception of the plateau region. The contact angle was measured using the Owen–Wendt Rabel and Kaelble method to confirm the surface energy of samples (I) and (II). Measurements of the advancing liquid–solid contact angle (θ) using water and diiodomethane were used to investigate the heterogeneity of the surface energy between the two samples, utilizing Young’s equation (Equation (1)) and Owen–Wendt equation (Equations (2) and (3)) [36].

(1) $$\gamma_{SG}=\gamma_{SL}+\gamma_{LG}\cos\theta$$

(2) $$\gamma_{SG}=\gamma_{SL}+\gamma_{LG}-2\sqrt{\gamma_{SG}^{d}\;\cdot \;\gamma_{LG}^{d}}-2\sqrt{\gamma_{SG}^{p}\;\cdot \;\gamma_{LG}^{p}}$$

(3) $$\gamma_{SG}{}_{total}=\gamma_{SG}{}_{}^{d}+\gamma_{SG}{}^{p}$$

where $\gamma_{SL}$ is the solid–liquid tension, $\gamma_{SG}$ is the solid-gas surface energy, $\gamma_{LG}$ is the liquid–gas interfacial energy, and θ is the contact angle. ‘*p*’ is the polar part and ‘*d*’ represents the dispersed part.

The surface energy is mainly determined by the surface roughness and qualities of the deposition layers, such as the binding mechanism to the surface, surface coverage, and molecular orientation [37,38]. Using the AFM measurements shown in Figure 3c,d, the surface roughness of sample (I) and (II) used in this study were measured to be 0.09 and 0.13 nm (RMS), respectively, and it was observed that the surface roughness of the two samples was similar. The contact angles of the water and diiodomethane droplets obtained from the top images of Figure 3e,f were verified. The surface energies were calculated using Equation (3), and the total surface energy values of samples (I) and (II) were 50.1 and 42.6 mN/m, respectively. Sample (I) was observed to have a higher total surface energy, as well as dispersed (non-polar) and polar components, when compared with that of sample (II); i.e., AlN/sapphire in sample (I) has a higher surface energy than the AlN/${\mathrm{SiO}}_{2}$/sapphire in sample (II), and the surface energy of the AlN layer located in the plateau region is higher than that of the AlN layer deposited on ${\mathrm{SiO}}_{2}.$ The variation in surface energy with the growth region is one of the factors influencing the preference for the initial growth of GaN, even though the GaN nucleation happened on the entire AlN layer. Table 1 summarizes the surface energies and roughness of the two samples.

**Figure 4:**
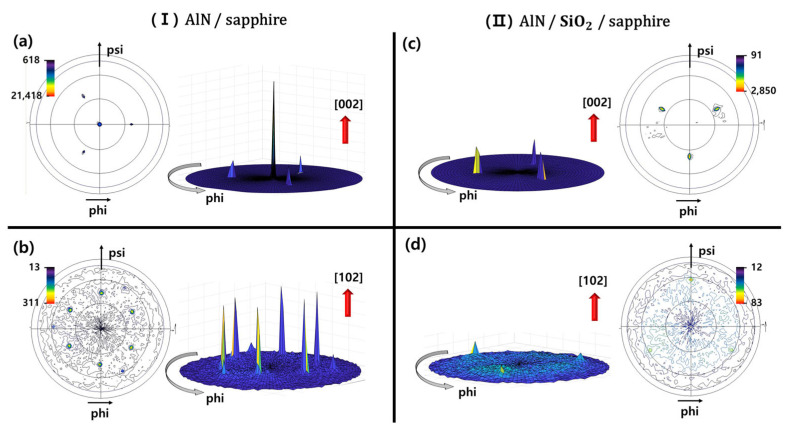
XRD 2D, 3D pole figure patterns obtained for (**a**) AlN (002) and (**b**) AlN (102) from (I) AlN/sapphire, and (**c**) AlN (002) and (**d**) AlN (102) from (II) AlN/${\mathrm{SiO}}_{2}$/sapphire. Both samples were heat-treated by raising the temperature to the GaN growth temperature.

The in-plane orientational difference between the AlN buffer layers on the sapphire and ${\mathrm{SiO}}_{2}$ substrates was verified using XRD pole-figure measurements. Figure 4 shows the XRD pole figures obtained for AlN (002) and AlN (102), plotting the 2D and 3D images of the samples, respectively: (I) AlN/sapphire and (II) AlN/${\mathrm{SiO}}_{2}$/sapphire. Figure 4a shows the XRD pole figure obtained for AlN (002) from sample (I). A strong center pole was detected in the AlN (002) poles, suggesting that the AlN buffer layer was deposited along the c-axis on sapphire, similar to epitaxial growth. The small intensity of the three symmetrical poles separated by 120° (ϕ) indicate sapphire (104), which has a similar d-spacing to AlN (002). The pole figure obtained for AlN (102) from sample (I) is depicted in Figure 4b and illustrates the in-plane relationship. The AlN (102) poles appear at six symmetrical positions separated by 60° (ϕ) at ψ = 42.5°. Clear evidence of the c-axis-oriented deposition of the AlN thin film was provided by the six very strong symmetrical poles observed for AlN (102). The three negligibly weak symmetrical poles separated by 120° (ϕ) of sapphire (024) were observed, which had a similar d-spacing to AlN (102).

**Figure 5:**
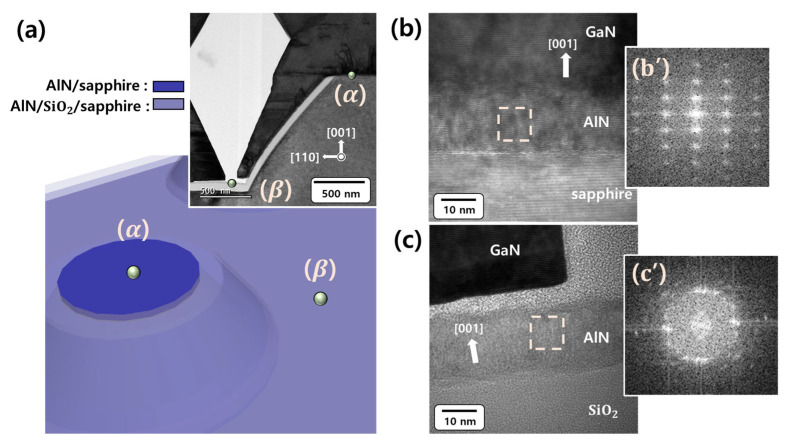
(**a**) A schematic representation of the plateau region and other regions on the PP-PSS and cross-sectional of low magnification TEM image of the mask layer in the PP-PSS. Cross-sectional of high magnification TEM image of the interface formed (**b**) between GaN and the substrate in the plateau region on the PP-PSS surface in the (α) position and (**c**) between AlN and ${\mathrm{SiO}}_{2}$ on the flat surface in the $\left(\beta \right)$ position; (**b’**,**c’**), the fast Fourier transform images of the intermediate AlN layers.

Contrastingly, Figure 4c,d show the XRD pole figures obtained for AlN (002) and AlN (102) from sample (II). The central pole or six symmetrical poles were not detected in any except three of the symmetrical poles, which indicate sapphire (104) and sapphire (024). The absence of these poles, with the exception of the pole of the sapphire substrate, indicates that the AlN thin film was deposited with no preferred in-plane orientation owing to the amorphous ${\mathrm{SiO}}_{2}$ layer, which means that a non-crystalline structure was present. When comparing samples (I) and (II), the AlN layer deposited on the sapphire substrate exhibits a c-axis crystal orientation similar to that of a single crystal through the pole figures observed for AlN (002) and AlN (102). However, the AlN layer deposited on ${\mathrm{SiO}}_{2}$ was non-crystalline. Despite depositing the AlN layer under the same conditions, the crystallinity was consistent with the difference in the polar component of the contact angle. Similar results were obtained for the indirect samples, as shown in Figure 5, even in the actual PP-PSS.

The schematic illustration in Figure 5a shows the PP-PSS deposited with an AlN layer prior to the GaN microrods being grown. The upper-right corner of Figure 5a shows the cross-sectional TEM image of the GaN [1$\bar{2}$0] zone axis, obtained by measuring the side of the PP-PSS on which the GaN microrods were grown. Although it is an AlN layer deposited under the same deposition conditions, it is marked with different colors to distinguish the AlN layers in the plateau region and ${\mathrm{SiO}}_{2}$ mask regions named (α) and (β). In Figure 5b,c, the high magnification TEM images of the GaN [1$\bar{2}$0] zone axis of position (α) and (β) marked in the PP-PSS in Figure 5a can be found. The TEM image of Figure 5b shows the GaN/AlN/sapphire interface at the plateau region of the PP-PSS, marked as (α) in the PP-PSS in Figure 5a. This can be confirmed through the well-aligned atomic arrangement in which the sapphire substrate is a single crystal. Additionally, some dislocations can be observed at the interface between GaN and AlN. However, a well-aligned GaN atomic arrangement can be seen, with the exception of the interface formed between GaN and AlN, indicating that GaN grew as a single-crystalline structure. The d-spacing of GaN (002) was 0.262 nm. The hexagonal AlN discrete diffraction spots indicate the growth of a single-crystalline-like AlN layer at the plateau region of the PSS surface, as shown in the fast Fourier transform (FFT) pattern in Figure 5(b’). However, the discrete diffraction spots appear to be slightly broad, which was consistent with the fact that some of the AlN interlayer atoms were arranged in a disordered manner, as shown in Figure 5b. Figure 5c shows the high-resolution TEM image of the AlN/${\mathrm{SiO}}_{2}$/sapphire interface located between the PP-PSS lens and the lens indicated by (β) in Figure 5a. The ${\mathrm{SiO}}_{2}$ layer was amorphous based on the atomic arrangement shown in Figure 5c. The atomic arrangement of AlN deposited on the ${\mathrm{SiO}}_{2}$ layer exhibits different growth orientations for each grain. In the fast Fourier transform (FFT) pattern observed between the corresponding AlN interlayers in Figure 5(c’), the spots associated with the in-plane direction were displayed in a nearby ring pattern, indicating that each AlN column was aligned in various in-plane orientations. Therefore, it can be concluded that the AlN on the ${\mathrm{SiO}}_{2}$ layer did not grow epitaxially. This confirmed that the crystallization results obtained at positions (α) and (β) on the PP-PSS, measured using TEM in Figure 5, were consistent with the pole figure results of samples (I) and (II) on c-plane sapphire, which are the indirect samples shown in Figure 4.

**Figure 6:**
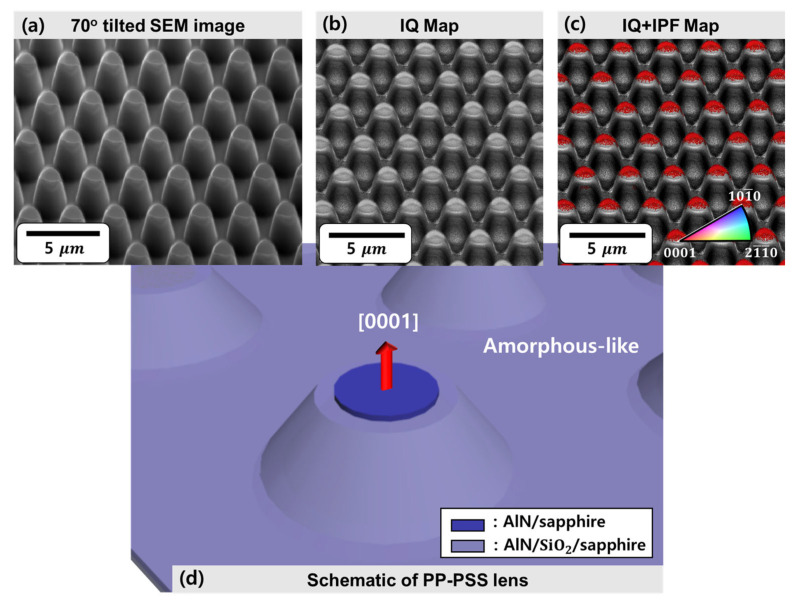
(**a**) The 70° tilted SEM image of PP-PSS, (**b**) image quality (IQ) map of PP-PSS using backscatter electron (BSE) diffraction, (**c**) EBSD image of the PP-PSS expressed by overlapping IQ and inverse pole figure (IPF) map, and (**d**) a schematic representation of the crystal orientation of the plateau region and other regions on the PP-PSS.

The growth orientation and crystallinity of the entire AlN layer on the PP-PSS were investigated using electron backscatter diffraction (EBSD), as shown in Figure 6. EBSD is a well-established technique for crystal orientation that quantifies the crystal phases and grain boundaries. We prepared a PP-PSS template, in which a 20 nm AlN layer was deposited for EBSD measurements. The prepared sample was loaded into the EBSD instrument at an inclination of ~70° to the normal of the incident electron beam. The SEM images of the PP-PSS obtained by tilting at 70° can be confirmed in Figure 6a. The SEM measurement range was set at 19 × 19 ${\;\mu m}^{2}$ and the high-angle SEM image was measured, which confirmed that the plateau region has a non-circular shape. Figure 6b shows the image quality (IQ) map of the PP-PSS constructed using backscattered electron (BSE) diffraction. The measurement range of the BSE mode was 19 × 19 ${\;\mu m}^{2}$. Additionally, Figure 6c shows the normal-direction EBSD inverse pole figure (IPF) map in Figure 6b, and is shown in alignment with the IQ map. All of the AlN layers located in the plateau region exhibit a red color in the normal-direction IPF map. However, no color was exhibited in the AlN layer deposited on the ${\mathrm{SiO}}_{2}$ substrate, other than on the plateau region. However, it can be seen that the AlN layer on the plateau region indicated by (I) AlN/sapphire in Figure 4 was deposited along the c-axis direction, whereas it can be observed that the AlN layers in the area other than the plateau region indicated by (II) AlN/${\mathrm{SiO}}_{2}$/sapphire in Figure 4 were not crystalline, as shown in Figure 6d. The results obtained for the crystal orientation of the AlN layer on the PP-PSS obtained using EBSD measurements were consistent with the pole figure measurements obtained for the indirect samples shown in Figure 4. This also indicates that the combination of EBSD and SEM is an effective tool for investigating the structural properties of individual microstructures on a large scale.

## 4. Conclusions

This study presented the experimental evidence for the mechanism of the GaN selective-area growth on a deposited template under the same deposition conditions using PP-PSS and indirect samples [(I) AlN/c-plane sapphire and (II) AlN /${\mathrm{SiO}}_{2}$/c-plane sapphire]. GaN microrods were successfully grown during 2, 4, 6, 10, and 100 cycles using pulsed-mode MOCVD on the PP-PSS, on which an AlN buffer layer was deposited. The GaN growth behavior during all cycles was found to selectively grow in the flat plateau region at the top of the PSS lens, instead of the total area of the PP-PSS, where all of the AlN layers were deposited. To confirm the reliability of the GaN selective-area growth on PP-PSS, a ${\mathrm{SiO}}_{2}$ layer was first deposited on half of the PP-PSS prior to the deposition of AlN, and GaN was then grown via MOCVD after the AlN layer was deposited, as an additional experiment. Although GaN was only selectively grown on the AlN layer of PP-PSS, where the plateau region was exposed to sapphire, it was confirmed that GaN grew in a disorderly manner in the AlN layer of PP-PSS, where ${\mathrm{SiO}}_{2}$ was deposited in all regions. The two indirect samples were characterized using contact angle, AFM, and pole figure measurements. The surface roughness values of samples (I) and (II) were similar; however, the surface energies were different (50.1 and 42.6 mN/m, respectively). In the pole figure results used to confirm the AlN crystal characteristics of the two samples, the AlN of sample (I) exhibited a c-axis orientation, whereas that of sample (II) exhibited no crystallinity. The pole figure results and the polar part of the surface energy are seen to match. TEM measurements of the region of PP-PSS, in which the GaN microrods were grown, showed that the AlN layer in the plateau region exhibited a single crystal structure. In contrast, the AlN layer in the area where ${\mathrm{SiO}}_{2}$ was deposited was similar to that of the amorphous AlN. Furthermore, by measuring the AlN-deposited PP-PSS using EBSD, the AlN along the c-axis was only detected in the plateau region, and no other crystalline AlN was detected even though AlN was deposited in its vicinity. This result is similar to the pole figure results of the indirect samples. Therefore, it was confirmed that the selective growth of GaN on the AlN buffer layer deposited under the same conditions is closely related to the surface energy and crystallinity.

## Figures and Tables

**Table 1:** Advancing contact angle observed for water and diiodomethane on (I) AlN/sapphire and (II) AlN/${\mathrm{SiO}}_{2}$/sapphire. The RMS of the surface roughness determined using AFM is also given.

		Contact Angle, θ [ °, Average]		Surface Energy [mN/m]		
Sample	RMS (n m)	Water $\left(H_{2}O\right)$	Diiodo-methane $\left(CH_{2}I_{2}\right)$	Disperse Component $\left(\gamma_{SG}^{d}\right)$	Polar Component $\left(\gamma_{SG}^{p}\right)$	Surface Energy $\left(\gamma_{SG}\right)$
(I) AlN/sapphire	0.09	58.2	49.5	34.5	15.5	50.1
(II) AlN/${\mathrm{SiO}}_{2}$/sapphire	0.13	68.2	54.8	31.6	11.0	42.6

## Data Availability

Not applicable.
